# Changes in the Hydrocarbon Proportions of Colony Odor and Their Consequences on Nestmate Recognition in Social Wasps

**DOI:** 10.1371/journal.pone.0065107

**Published:** 2013-05-29

**Authors:** Elena Costanzi, Anne-Geneviève Bagnères, Maria Cristina Lorenzi

**Affiliations:** 1 Department of Life Sciences and Systems Biology, Università di Torino, Torino, Italy; 2 I.R.B.I. – UMR CNRS 7261 – Université de Tours, Faculté des Sciences, Tours, France; Université Paris 13, France

## Abstract

In social insects, colonies have exclusive memberships and residents promptly detect and reject non-nestmates. Blends of epicuticular hydrocarbons communicate colony affiliation, but the question remains how social insects use the complex information in the blends to discriminate between nestmates and non-nestmates. To test this we altered colony odor by simulating interspecific nest usurpation. We split *Polistes dominulus* paper-wasp nests into two halves and assigned a half to the original foundress and the other half to a *P. nimphus* usurper for 4 days. We then removed foundresses and usurpers from nests and investigated whether emerging *P. dominulus* workers recognized their never-before-encountered mothers, usurpers and non-nestmates of the two species. Behavioral and chemical analyses of wasps and nests indicated that 1) foundresses marked their nests with their cuticular hydrocarbons; 2) usurpers overmarked foundress marks and 3) emerging workers learned colony odor from nests as the odor of the female that was last on nest. However, notwithstanding colony odor was usurper-biased in usurped nests, workers from these nests recognized their mothers, suggesting that there were pre-imaginal and/or genetically encoded components in colony-odor learning. Surprisingly, workers from usurped nests also erroneously tolerated *P. nimphus* non-nestmates, suggesting they could not tell odor differences between their *P. nimphus* usurpers and *P. nimphus* non-nestmates. Usurpers changed the odors of their nests quantitatively, because the two species had cuticular hydrocarbon profiles that differed only quantitatively. Possibly, *P. dominulus* workers were unable to detect differences between nestmate and non-nestmate *P. nimphus* because the concentration of some peaks in these wasps was beyond the range of workers' discriminatory abilities (as stated by Weber's law). Indeed, workers displayed the least discrimination abilities in the usurped nests where the relative odor changes due to usurpation were the largest, suggesting that hydrocarbon variations beyond species-specific ranges can alter discrimination abilities.

## Introduction

Interactions among animals, like mate choice or cooperative behaviors, often occur after individuals recognize each others. In social groups, the ability to recognize nestmates and discriminate them from non-nestmates is crucial for group integrity and is favored by selection [1]. Nestmate recognition is often based on a complex process of phenotype matching, where interacting individuals compare phenotypic traits of the unidentified individuals with a neural template [2]. Phenotype matching is therefore a perceptual process (a sort of stimulus generalization) in which an animal's response to a stimulus depends on its similarity/dissimilarity to a neural template [2]. Templates may be genetically encoded [3], [4] and/or acquired through a learning process [5]. Animals usually acquire templates when they are more likely to live in close association with kin, e.g., during ontogeny at natal nests. Then, they store the information in their memories. For example, long-tailed tits learn their siblings' calls when they are young and later discriminate kin from non-kin on the basis of their vocalizations [6]. Similarly, Belding's ground squirrels learn their sibling scents at natal nests and later discriminate kin from non-kin on the basis of scents, although they also seem to use their own cues [7] or genetically encoded templates to recognize unfamiliar kin [8].

Social insects live in complex societies, with exclusive colonies where nestmates are the only admitted members [9]. With a few exception [10], exclusiveness is a crucial property in social insect organization, as nestmate/non-nestmate discrimination delimit colonies, protect them from robberies and prevent workers from helping unrelated, parasitic intruders at a cost for workers' relatives. Nestmate/non-nestmate discrimination may involve multiple sensory channels [11], [12], but ants, bees wasps and termites use mainly chemical codes to communicate colony affiliation securely. The neural recognition templates of social insects are thought to form by smelling the Gestalt odor of their colonies as soon as they emerge [13], [14], [15] and by storing it in experience-derived memory. Later, colony residents will match the odor of unidentified individuals to the acquired template. The decision to accept or reject is modeled by algorithms [2], [16]. Individuals are admitted to a colony if their odors match the residents' neural templates; they are rejected if it does not. Colony residents change their behavioral responses from admission to rejection when the dissimilarity between their neural template and the odor of unidentified individuals exceeds the acceptance threshold [2]. The detection of dissimilarities between the chemical profiles of unidentified individuals and the neural templates is therefore crucial to discrimination processes.

In paper wasps, colony odor is foundress-derived: foundresses mark their paper nests with their own odor [17]. Paper wasp colonies are often usurped by facultative social parasites (thereafter, usurpers) [18], [19]. Usurpers are foundresses of free-living species that usurp colonies of the same or, more rarely, of other species. Usurpers exploit nests as well as workers of the displaced females for their own reproduction [19]. Usurpers overmark the foundresses' odor marks [17] by stroking their abdomens on the nest surfaces [20], [21]. When host workers emerge in usurped nests, they learn the usurper-odor marks as their colony odor and therefore accept their usurpers as nestmates. This ensures that host workers (i.e., genetic daughters of the displaced foundresses) direct their cooperative behaviors to the unrelated usurpers (and their brood).

Colony odors in social insects are complex, usually species-specific, mixtures of up to 100 different hydrocarbons that constitute the cuticular chemical profiles of insects [22]. The relative proportions of hydrocarbons vary between individuals, but variations are smaller between individuals from the same colonies than from different colonies [23], [24], [25]. Presumably, the detection of these differences allows for nestmate/non-nestmate discrimination. However, social insects often accept unidentified individuals that have low hydrocarbon concentration (irrespective of differences), possibly because their identification is more difficult when recognition cues are not enough. For example, social insects do not attack dead non-nestmates when their cuticular hydrocarbons have been washed out [23], [25]. Additionally, workers spend less time attacking lures with low hydrocarbon concentrations [26]. Finally, social parasites often have lower concentrations of cuticular hydrocarbons than their hosts, indirectly supporting the hypothesis that the lack of hydrocarbons facilitates acceptance [13], [27], [28], [29], [30]. All these observations suggest that intruders that possess few recognition cues do not trigger aggression.

Cross-fostering experiments may be used to identify whether quantitative hydrocarbon variations are involved in the recognition process. In these experiments, insects face natural changes in colony odors involving naturally occurring compounds in natural concentration ranges [17], [31], [32], [33], although it may be difficult to understand how colony odor changes (in terms of overmarks blending in, staying distinct and/or masking the original marks) [34].

We studied how usurpers alter colony odors in paper-wasps to understand to what extent the variation in the hydrocarbon ratio alter nestmate/non-nestmate discrimination, using *P. nimphus* and *P. dominulus* (two closely related species) [35], [36]. Behavioral analyses indicated that *P. nimphus* usurpers overmarked the foundress marks when they usurped *P. dominulus* colonies [33]. Preliminary chemical analyses indicated that *P. nimphus* and *P. dominulus* wasps had similar chemical composition of cuticular hydrocarbon blends, but distinct relative proportions of hydrocarbons (this paper). This gave us the chance to manipulate *P. dominulus* colony odors by changing the relative proportions of hydrocarbons. We performed a sort of cross-fostering experiment, where *P. dominulus* brood emerged in nests marked by their genetic mothers or overmarked by *P. nimphus* usurpers.

Usurpers usually invade conspecific nests (intraspecific usurpation); few reports exist where usurpers invade the nests of another species (interspecific usurpation) [37]. Among these rare reports, *P. nimphus* wasps were found as usurpers in *P. dominulus* colonies [21].

We used here *P. nimphus* females as usurpers in *P. dominulus* colonies. In order to control for colony-specific trait variations in workers, including those involved in recognition processes (e.g., perceptual threshold levels, discrimination accuracy, aggression thresholds and olfactory experience when larvae), we cut each *P. dominulus* nest into two parts. We put a half-nest in the *P. dominulus* foundress care and the other half in the *P. nimphus* usurper care. We expected that foundresses marked their nests and usurpers overmarked foundress marks. Emerging *P. dominulus* workers would learn either the foundress marks or the usurper overmarks, depending on where they emerged. Therefore, some workers would learn a “regular” *P. dominulus* template, whereas their cross-fostered sisters would learn a “*P. nimphus*-biased” template. We expected that the scent marks of the two nest parts would differ only in their relative proportions of hydrocarbons, because the hydrocarbon profiles of the two species differed quantitatively, not qualitatively. Finally, we tested how the variation in the relative proportions of the hydrocarbons in colony odors affected nestmate/non-nestmate discrimination.

## Materials and Methods

### Ethic Statement

The collection of colonies and the experiments performed comply with the current laws in Italy. No specific permits were required for the collection neither for collection location. The species used in the experiments were not endangered or protected in Italy.

### Behavioral analysis

#### Nest collection, rearing and cutting

We collected 41 *P. dominulus* and 46 *P. nimphus* singly-founded colonies in the pre-emergence phase from areas in North-West Italy: Monforte d'Alba (Cuneo), Settimo Torinese and Orbassano (Torino). In these areas the two species were sympatric. We choose 14 *P. dominulus* colonies for the nest-splitting experiment (nests were large and symmetrically shaped) and 14 *P. nimphus* foundresses as usurpers. The other *P. dominulus* and *P. nimphus* foundresses were killed by freezing and stored at −18°C to be used later as non-nestmates in recognition tests.

**Figure 1:**
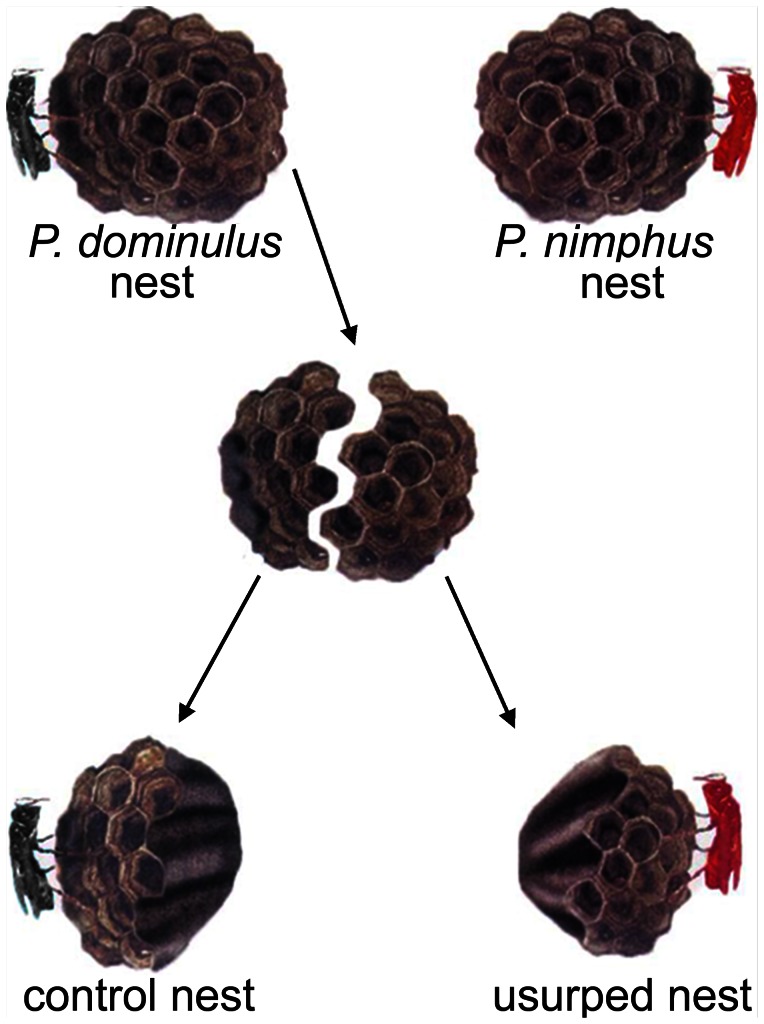
Nest cutting. The experimental procedure of cutting *P. dominulus* nests into two parts and assigning a half to the original foundress (in black) and the other to a *P. nimphus* foundress (in red) to obtain a control nest and a usurped nest.

In laboratory, the 14 P. *dominulus* nests were cut into halves with clean scissors (Fig. 1). During nest cutting, foundresses were kept in glass jars. The 28 half-nests were separately placed in glass boxes (15×15×15 cm). Then, we introduced into each box either the original *P. dominulus* foundress or a *P. nimphus* foundress. In the laboratory, paper-wasp foundresses readily adopt foreign colonies and behave as usurpers [17], [33]. Therefore, a half nest was reared by its own foundress (control nest) and the other half by a *P. nimphus* foundress (usurped nest). Colonies were supplied with water, honey and *Tenebrio molitor* larvae *ad libitum*. The cages were kept at room temperature (26–28°C) under 12 L/D artificial illumination (100 W bulbs).

The two halves contained similar numbers of pupae (mean±S.E., in control nests: 2.20±0.49; in usurped nests: 3.10±0.31; Wilcoxon test, Z = −1.628, P = 0.103).

Foundresses and usurpers spent 4 days on their nests (previous experiments documented that usurpers successfully overmarked foundress marks in 4 days) [33]. Then, they were removed, killed by freezing and stored at −18°C until they were used for behavioral tests and chemical analyses.

Workers emerged after we removed foundresses or usurpers (a few workers emerging before were eliminated). All workers were likely genetic daughters of the foundresses, because nests were originally singly-founded and all age-classes of immature brood were present (this is an indication that no usurpation occurred since usurpers eliminate eggs and small larvae of the displaced foundresses) [38]. We performed behavioral and chemical tests on a total n = 20 nests where usurpers successfully adopted the nests and emerging workers did not desert.

#### Behavioral tests

We tested how *P. dominulus* workers from usurped nests changed their responses to their own foundress, their *P. nimphus* usurper, and to *P. dominulus* and *P. nimphus* non-nestmates as compared to the responses of their sisters in control nests. We avoided behavioral or chemical interference by the wasps used as stimuli because they were dead (dead insects have been routinely used for recognition tests) [17], [23], [25], [39], [40]; and the hydrocarbon blends of dead insects are stable over time [41].

Workers had never met their foundresses or usurpers before behavioral tests. However, these workers were living on their nests. *Polistes* wasps exposed to nest (or nest fragments) learn to recognize nestmates [42], whereas those exposed to nestmates do not [43]. Behavioral tests were performed on workers >24 hours (wasps learn their colony odor within few hours from emergence) [44] that had similar ages and were in similar numbers in control and usurped nests (mean±S.E.; age of workers in control nests: 2.1±0.5 days; usurped nests: 1.5±0.2 days; Wilcoxon test: Z = −0.843, P = 0.399; number of workers in control nests: 2.00±0.33; usurped nests: 2.00±0.21; Wilcoxon test, total n = 18; Z = −0.333, P = 0.739).

We introduced the four stimulus wasps (foundress, usurper or the two kinds of non-nestmates) into each nest one at a time, in random order, at intervals of at least 30 min. We hold the stimulus wasps by forceps in front of the nest (at a distance of 1 cm from the anterior face of the nest). Each test lasted 1 min since workers had exhibited the first response towards the stimulus wasp (i.e., inspection, antennal contact or any other behavior).

The observer was blind to wasp affiliation, and partially blind to the species (*P. dominulus* and *P. nimphus* only differ macroscopically in the color of the 6^th^ abdominal sternum) [45].

During the tests, the observer counted the number of intolerant behaviors that workers exhibited towards the stimulus wasps (bites, attacks, leaving the nest, grasping, and stings). Behavioral tests were performed as soon as workers emerged and were >24 hours old, which occurred within two weeks since we introduced usurpers.

For the analyses of data, we divided the number of intolerant behaviors counted in a given half-nest by the number of workers that were on that half-nest during the test (thereafter “attacks”).

Following Gamboa [14], we interpreted nestmate recognition as the differential responses that workers exhibited to the wasp used as stimulus, where reduced aggression meant tolerance of nestmates and increased aggression meant rejection of non-nestmates.

We expected that workers from usurped nests would attack usurpers less often than their sisters from control nests, if usurpers marked the nests. In this case, we also expected that worker in usurped nests would attack their foundresses, if usurper overmarks masked the foundress marks. Alternatively, in case usurpers chemically mimicked their usurped nest odors, workers from both usurped and control nests would have accepted them. We also expected that workers attacked non-nestmates of both species in both nest parts.

### Chemical analysis

#### Collection of epicuticular hydrocarbons

After the behavioral tests, we analysed the hydrocarbon profiles of the 10 foundresses, 9 usurpers (we accidentally lost one extract) and a 1-cm^2^ sample of the paper of their nests (n = 10 control nests and n = 10 usurped nests).

We weighed the wasps and the nest-paper samples with a precision balance Precisa 125A. We extracted the epicuticular hydrocarbons of the wasps and of the nest-paper samples by dipping each sample separately in 1 ml of pentane for 90 sec. Before analysis, we added 800 ng of *n*-eicosane (C20) to each extract as an internal standard.

#### Cuticular hydrocarbon analysis

For quantitative analyses, we analysed the samples using gas-chromatography with flame ionization detection (Delsi Nermag DN200). Two μl of each extract were injected splitless (15 sec) at 70°C in the GC programmed so that temperature increased to 150°C at a rate of 30°C/min. The 150°C temperature was kept for 5 min, and then increased to 320°C at a rate of 5°C/min. The GC was equipped with a non-polar Chrompack CPSIL5 WCOT CB column (25 m×0.25 mm×0.12 µm). Helium was the carrier gas (1 bar). Results were registered by a “Standard ChemStation” program (G170101AA, Version A.03.00, copyright © Hewlett-Packard 1989-1996).

For identification of compounds, we analysed the extracts by gas-chromatography/mass spectrometry (GC/MS) (Hewlett Packard GC 5890 coupled with MS Engine Hp 5989A); the entire system was controlled by a MS Chemstation. The analyses were run at 70 eV (electronic impact, EI). Mass spectra were obtained in the following conditions with m/z 45-500: 1 scan/sec, source temperature 250°C, quadrupole 100°C, interface 300°C. The capillary column and the temperature program were the same as those used for GC. After the identification of the compounds, we calculated the relative abundance of each compounds by an automatic function of the program “Standard ChemStation”.

#### Statistical analyses

Our experiment had a matched-subjects design because data were inherently paired (pairs of nests, mothers/usurpers that were last on matched nests, sisters from matched nests) [46]. Therefore, we used Wilcoxon tests or repeated measures GLM to analyze differences between matched pairs of data, depending on whether data accounted for normality and homoscedasticity assumptions.

In behavioral analyses, the mean numbers of attacks per worker were ln-transformed to account for normality and homoscedasticity assumptions.

We checked whether usurper had successfully changed the colony odor of usurped nests. Since it was impossible to include all the identified compounds in the analyses (>60 hydrocarbons), we excluded from the whole data set 6 compounds <3%. We further reduced the number of variables by performing two separate Principal Component Analysis (PCAs), one on branched alkanes and linear linear alkenes (43 variables) and another on linear alkanes (12 variables) (PCAs were based on correlations, varimax rotation). For each analysis, we re-computed the relative proportions of hydrocarbons, then transformed these compositional data by using the log-ratio-transformation (natural log of the proportion of each peak divided by the geometric mean of the proportions of linear alkanes) [47]. The PCA on the branched alkanes and linear alkenes produced 7 principal components (eigenvalues > 1; variance explained 85.30%). The PCA on linear alkanes produced 4 principal components (eigenvalues > 1; variance explained 82.46%). These components were analysed by using two separate stepwise DAs. In the DAs, the grouping variable was the nest part (n = 20 samples for the foundress part and n = 19 samples for the usurper part) and the independent variables were the principal component values (within-group covariance matrix; Mahalanobis distance method).

We expected that the DAs significantly discriminated whether a sample belonged to the foundress or usurped part, if foundresses and usurpers had marked their nests with their own odor.

We also measured the chemical similarity (euclidean distances, Z-scores transformed values) between the female that was last on the nest and the nest itself (i.e., foundresses and their nests or usurpers and their nests).

Following Lenoir et al. [13], we measured the concentration of hydrocarbons in each extract as the sum of all peak areas divided by the area of *n*-C20 (which amounted to 800 ng) and by correcting for weight (ng of hydrocarbons/mg of insect body weight or nest-paper weight).

Finally, we tested whether changes in colony odor affected the discrimination abilities in the usurped workers. Specifically, for the hydrocarbons with high loadings on the Principal Components, we calculated the differences in hydrocarbon concentration between each usurped nest and the relative control nest. Then, we tested whether significant increases in the hydrocarbon concentrations affected worker discrimination abilities (we were interested in increases in hydrocarbon concentration because decreases in recognition cues do not usually trigger aggression in social insects - see introduction section). We tested the directional hypothesis that increases in colony odor negatively affected worker discrimination abilities, using a one-tailed Spearman correlation test. We did not apply Bonferroni corrections so as to avoid over-inflation of Type II error [48].

Descriptive statistics are mean±S.E. Statistical analyses were performed in SPSS Statistics 18.0. SIMPER similarities were computed in PAST (Paleontological Statistics) [49].

## Results

### Behavioral tests

#### Workers from usurped nests tolerated usurpers

**Figure 2:**
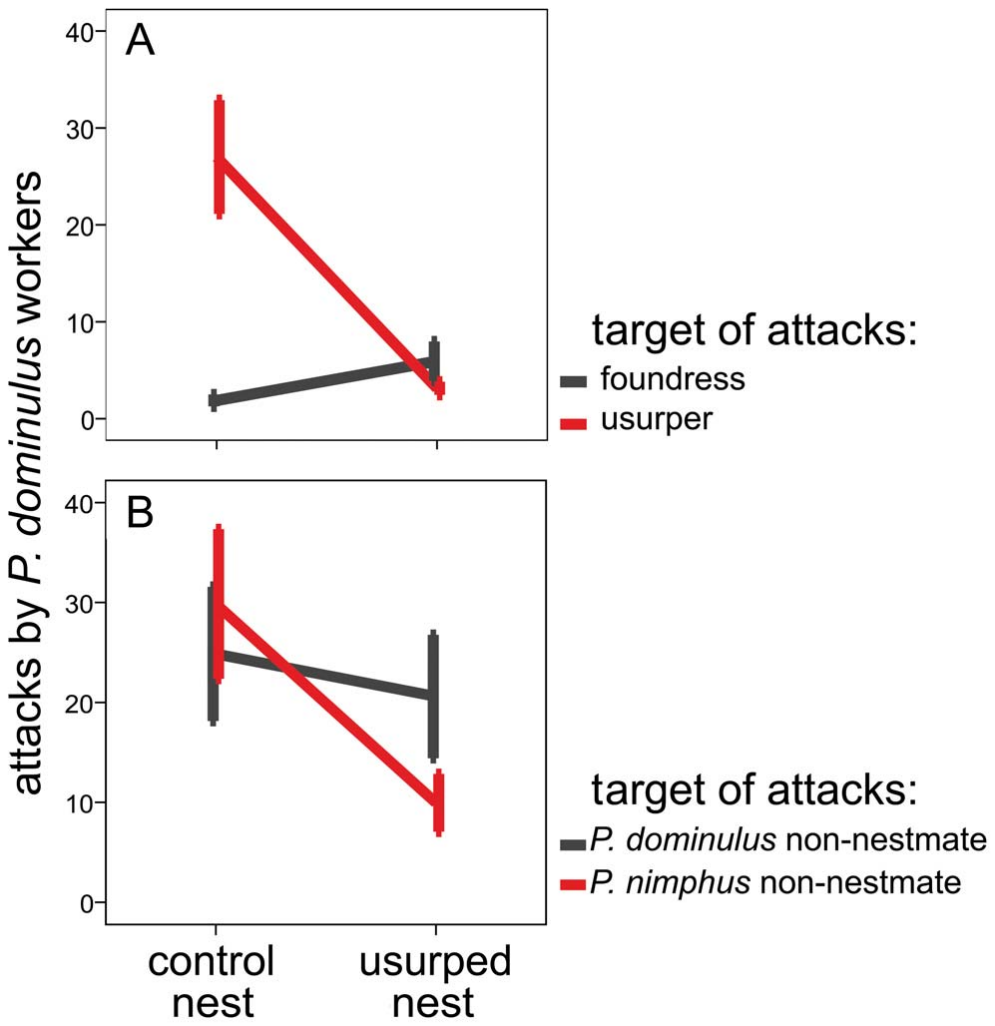
Behavioral tests. The results of the behavioral tests as the mean number of attacks (± SE) that *P. dominulus* workers displayed against the stimulus wasps. A) Workers from the two nest parts responded differentially to foundresses and usurpers – attacks to usurpers (but not foundresses) dropped in usurped nests, relatively to control nests - and (B) sisters from the two nest parts responded differentially to the non-nestmates of the two species - attacks to *P. nimphus* non-nestmates (but not those to *P. dominulus* non-nestmates) dropped in usurped nests, relatively to control nests.

Relative to their sisters in control nests, the workers in usurped nests changed significantly their responses depending on whether they responded to usurpers or foundresses (repeated measures GLM: interaction nest * target species: Wilk's λ = 0.716, F_1,16_ = 34.561, P<0.0001) (Fig. 2A). The mean number of attacks to usurpers in control nests was 27.00±5.87, which dropped to 3.15±0.68 in usurped nests. The increased tolerance towards usurpers in usurped nests indicated that usurpers marked their nests. In contrast, the mean number of attacks to their own foundresses was low in both nest parts (control nests: 1.89±0.61; usurped nests: 5.95±2.01) (Fig. 2A).

#### Workers from usurped nests were aggressive towards *P. dominulus* non-nestmates but tolerant towards *P. nimphus* non-nestmates

Relative to their sisters in control nests, the workers in usurped nests changed significantly their responses to non-nestmates and the changes depended on whether the stimulus wasps were *P. nimphus* or *P. dominulus* non-nestmates (repeated measures GLM: interaction nest * target species: Wilk's λ = 0.779, F_1,16_ = 4.531, P = 0.049) (Fig. 2B). The mean number of attacks to *P. nimphus* non-nestmates in control nests was 29.86±7.50, which dropped to 9.95±2.90 in usurped nests. This variation did not occur towards *P. dominulus* non-nestmates, which received similar number of attacks in both nest parts (control nests: 24.86±6.72; usurped nests: 20.60±6.18) (Fig. 2B).

### Chemical analysis

#### The chemical profiles of *P. dominulus* and *P. nimphus*

**Figure 3:**
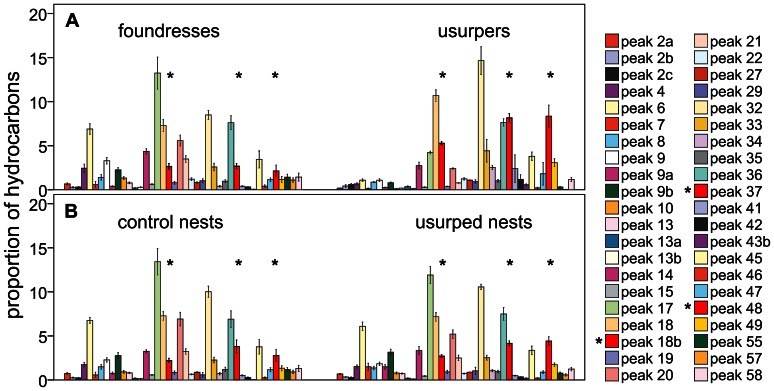
The proportions of branched alkanes and linear alkenes in A) foundresses, B) usurpers, C) control nests and D) usurped nests. Bars are mean (± E.S.) of proportions. The asterisks indicate the three peaks whose increases in usurped nests might have strong consequences on worker discrimination abilities (see text).

*P. dominulus* and *P. nimphus* wasps had complex cuticular hydrocarbon blends that included 63 identified hydrocarbons in 58 peaks; these were homologous series of linear alkanes, methyl-branched alkanes and linear alkenes between C_23_ and C_35_, as previously described (for *P. dominulus* see [50], [51]; for *P. nimphus* see [52]) (Table 1). The proportions of these hydrocarbons were species-specific (SIMPER similarity: 61.2%) (Fig. 3A). The nests had profiles relatively similar to those of the females that were last on the nests (see below; Fig. 3A, B).

**Table 1:** Cuticular hydrocarbons of *Polistes dominulus* and *P. nimphus*.

Peak	Hydrocarbon	Peak	Hydrocarbon
0	C23:1	25	*n*C30
1	*n*C23	26	Unknown
1b	*n*C24	27	16+15+14-MeC30
1c	2-MeC24	28	7-MeC30
2	*n*C25	29	2-MeC30
2a	13+11-MeC25	30	C31:1+Uk
2b	7-MeC25	31	*n*C31
2c	5-MeC25	32	15+13-MeC31
2d	3-MeC25	33	7-MeC31
3	*n*C26	34	5-MeC31+13,17-diMe+13,19-diMeC31
4	2-MeC26	35	11,17-diMeC31
5	*n*C27	36	7,15-diMeC31
6	13+11+9-Me27	37	5,15+5,19-diMeC31
7	7-Me27	38	Unknown
8	5-Me27+9,13-diMeC27	39	*n*C32+Unknown
9	2-MeC27	40	Unknown
9a	C28:1	41	16-MeC32
9b	3-MeC27	42	8-MeC32
10	5,15-diMeC27	43	2-MeC32
11	diMeC27	43b	C33:1
12	*n*C28	44	*n*C33
13	14+13+12-MeC28	45	17+15+13-MeC35
13a	7-MeC28	46	7-MeC33
13b	6-MeC28+5-MeC28	47	13,17-diMeC33+11,15+9,15-diMeC33
14	2-MeC28	48	7,17-diMeC33
15	C29:1	49	5,17-diMeC33
16	*n*C29	50	*n*C34+Unknown
17	15+13+11-MeC29	51	Unknown
18	7-Me29	52	16-MeC34
18b	5-MeC29	53	2-MeC34
19	11,17+11,15+11,13-diMeC29	54	*n*C35
20	9,17-diMeC29+3-MeC29+C30:1	55	17+15+13-MeC35
21	7,17-diMeC29	56	Unknown
22	5,17-diMeC29	57	13,17-diMeC35
23	Unknown	58	7,17+7,19-diMeC35
24	Unknown		

#### The chemical profiles of usurped nests were usurper-biased

The euclidean distances between usurpers and their own nests were significantly smaller than the distances between usurpers and matched control nests (mean euclidean chemical distances of usurpers *vs* own nests: 10.28±0.46; usurpers *vs* control nests: 12.11±0.61; Wilcoxon test, Z = −2.192, P = 0.028). This suggested that usurpers changed the chemical profiles of the usurped nests, making them more similar to their own profiles than their matched control nests were. Indeed, the euclidean distances between the foundresses and their own nests were significantly smaller than those between the foundresses and their usurped nests (mean euclidean chemical distances-foundresses *vs* own nests: 5.89±0.48; foundresses *vs* usurped nests: 7.41±0.60; Wilcoxon test, z = −2.191, P = 0.028).

#### The chemical profiles of nests, foundresses and usurpers

The chemical profiles of the control and usurped parts were significantly distinguished through the stepwise DA on the PCs of the linear alkanes. The solution that included just PC1 and PC3 as explanatory variables in the stepwise procedure offered the best discrimination between the nest parts (Wilks' λ = 0.381, d.f. = 2 P<0.0001), and 89.7% of the samples were correctly classified. This suggested that the linear alkanes that loaded the most on PC1 and PC3 might be the hydrocarbons that changed the most due to usurpation (r > 0.700, Table 2).

**Table 2:** The factor loadings.

	PCA on branched alkanes and linear alkenes				PCA on linear alkanes	
Peak	PC1	PC2	PC3	PC5	PC1	PC3
peak 37*	0.916					
**peak 49**	0.915					
**peak 2c**	0.911					
peak 18b*	0.909					
**peak 41a**	0.878					
**peak 21**	0.846					
**peak 45**	0.841					
**peak 33**	0.825					
**peak 32**	0.823					
**peak 18**	0.820					
**peak 2b**	0.815					
**peak 27**	0.782					
**peak 36**	0.719					
**peak 6**		0.958				
**peak 13a**		0.946				
**peak 20′**		0.901				
**peak 17**		0.895				
**peak 9b**		0.878				
**peak 4**		0.875				
**peak 10**		0.866				
**peak 20**		0.850				
**peak 9**		0.817				
**peak 15**		0.784				
**peak 2a**		0.764				
**peak 19**		0.708				
peak 48*			−0.775			
**peak 35**				0.888		
**peak 39**					0.911	
peak 50*					0.849	
peak 16*					0.842	
peak 1*						−0.879

The factor score loadings (> 0.700) of the peaks on the PCs used to derive the discriminant functions in the stepwise DAs. The PCs are sorted by their relative importance in the stepwise DAs. The peaks are sorted by loading size; high loadings indicated that the peak was highly correlated with the PC. *The asterisks highlight the discriminant peaks (see text).

**Figure 4:**
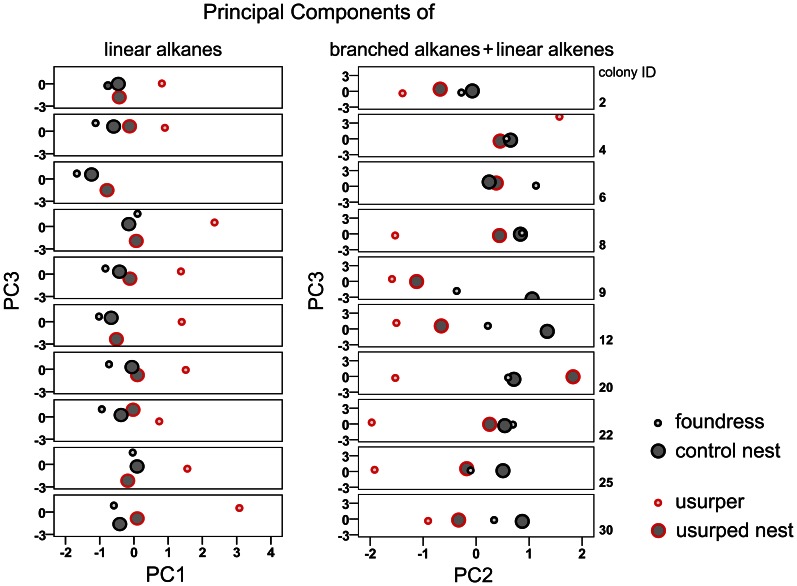
The changes in colony odor due to usurpation. Cuticular hydrocarbon variations in host colonies (colony ID: 2, 4, 6, 8, 9, 12, 20, 22, 25, 30). Each symbol represents either the foundress or the usurper or the control or usurped nests. The plots on the left show the projections of the two most discriminant components (PC1 and PC3) in the DAs based on the linear-alkane fraction. The plots on the right show the projections of the two most discriminant components (PC2 and PC3) in the DAs based on the branched-alkane and linear-alkene fraction.

The chemical profiles of the control and usurped parts were also significantly distinguished through the stepwise DA on the PCs of the branched alkanes and linear alkenes. Here, the solution that included, in order, PC2, PC3, PC5 and PC1 as explanatory variables offered the best discrimination between the nest parts (Wilks' λ = 0.482, d.f. = 4, P<0.0001), and 82.1% of the samples were correctly classified (Fig. 4). Therefore, the branched alkanes and linear alkenes that loaded the most on PC2, PC3, PC5 and PC1 might be the hydrocarbons that changed the most due to usurpation (r > 0.700, Table 2).

Therefore, the PCAs, DAs and the comparisons of Euclidean distances suggested that usurpers changed the concentrations of hydrocarbons in usurped nests.

#### The changes in hydrocarbon concentrations in usurped nests

Among the peaks with high loadings on the PCs (Table 2), usurped nests had significantly higher concentrations in peak 1, 16, 18b, 37, 39, 48 and 50 than control nests (thereafter, discriminant peaks, Table 3). Similarly, usurpers had higher concentrations in these peaks than foundresses, except for peak 1 (Table 3). However, the two nest parts had roughly equivalent overall concentrations of hydrocarbons because other hydrocarbons were less concentrated in usurped than in control nests (control nests: 1377.53±212.06 ng/mg of nest; usurped nests: 1423.61±80.33 ng/mg of nest, pairwise Wilcoxon test, Z = −0.153, P = 0.878). Similarly, the foundresses and the usurpers had roughly equivalent overall concentrations of epicuticular hydrocarbons (foundresses: 163.61±19.22 ng per mg of wasp; usurpers: 186.33±27.61 ng per mg of wasp, Z = −1.007, P = 0.314).

**Table 3:** The concentration of the discriminant peaks.

	mean concentration in control nests (ng/mg of nest)	mean concentration in usurped nests (ng/mg of nest)	pairwise Wilcoxon test		mean concentration in foundresses (ng/mg of wasp)	mean concentration in usurpers (ng/mg of wasp)	pairwise Wilcoxon test	
			Z statistic	*P* value			Z statistic	*P* value
**peak 1**	17.78±5.41	36.06±7.04	−1.988	**0.047**	0.55±0.18	0.93±0.21	−1.244	0.214
**peak 16**	72.09±11.24	104.57±8.01	−2.599	**0.009**	12.41±1.28	23.05±3.51	−2.429	**0.015**
**peak 18b**	20.57±3.16	29.98±3.51	−2.191	**0.028**	2.44±0.25	6.75±1.09	−2.666	**0.008**
**peak 37**	32.55±4.67	40.78±4.23	−.988	**0.047**	2.51±0.28	10.83±2.25	−1.988	**0.047**
**peak 39**	7.52 ±1.58	11.95±1.25	−.090	**0.037**	0.56±0.16	3.29±0.64	−.666	**0.008**
**peak 48**	26.53±7.28	42.29±4.50	−.395	**0.017**	1.97±0.57	9.06±1.71	−.547	**0.011**
**peak 50**	1.28±0.32	2.62±0.33	−.497	**0.013**	0.20±0.02	0.65±0.19	−.547	**0.011**

The concentration (in nests and wasps) of the peaks that increased significantly in usurped nests due to usurpation. Pairwise Wilcoxon statistics is also shown. Significant P values in bold.

#### Changes in colony odor and discrimination abilities

**Figure 5:**
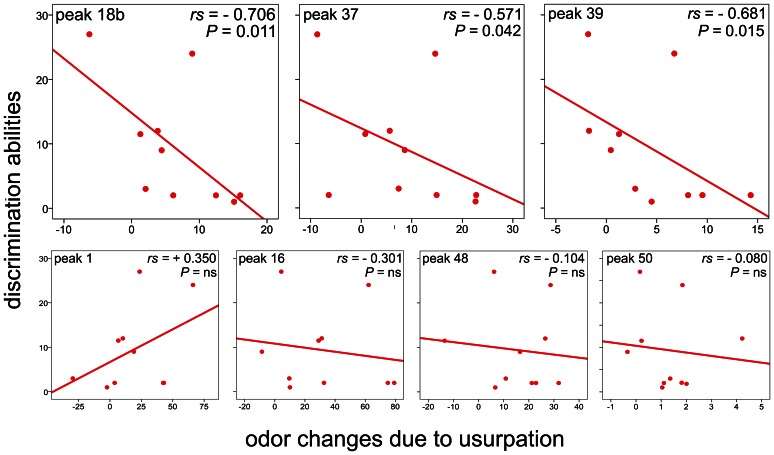
The relationship between discrimination abilities and odor changes due to usurpation. For peak 18b, 37 and 39, the correlation was significant: the discrimination abilities were the least where the relative odor changes due to usurpation were the largest. For peak 1, 16, 48 and 50, the correlation was not significant (discrimination abilities: attacks to *P. nimphus* non-nestmates; odor changes: difference between the concentration of the hydrocarbons in usurped and control nests; r = Spearman rho).

The least discrimination abilities occurred in colonies where the relative changes in the discriminant peaks were the largest (Fig. 5), suggesting that the large increases in the concentration triggered fewer attacks towards wasps of the usurper species. In contrast, more attacks were counted in colonies where these increases were not as large. Indeed, the increases in the concentration of three discriminant peaks (peak 18b, 37 and 39) due to usurpation were negatively and significantly correlated to worker discrimination abilities (Fig. 5). For the other discriminant peaks (peaks 16, 48 and 50) the increases in concentration were negatively correlated with discrimination abilities, but the correlations were non significant (peak 16: Spearman rho = −.301, P = 0.199; peak 48: rho = −.104; P = 0.387; peak 50: rho = −0.080, P = 0.413) Peak 1 was the only discriminant peak whose change in concentration was positively, although non significantly, correlated with discrimination abilities (rho = 0.350; P = 0.161). However, foundresses and usurpers did not differ significantly in the concentration of peak 1 on their cuticle, thus this peak cannot be used to test the relationship between odor changes and discrimination abilities.

#### Hydrocarbon concentrations varied between usurpers

All the peaks varied in concentrations among both foundresses and usurpers. Except for peak 1, the discriminant peaks had even larger variance in usurpers than in foundresses (Levene's test, peak 16: W_1,17_ = 8.010, P = 0.012; peak 18b: W_1,17_ = 28.623, P<0.0001; peak 37: W_1,17_ = 13.696, P = 0.002; peak 39: W_1,17_ = 5.459, P = 0.032; peak 48: W_1,17_ = 4.684, P = 0.045; and peak 50, W_1,17_ = 5.028, P = 0.039).

## Discussion

These results suggest that foundresses marked their nests with their cuticular hydrocarbons and usurpers overmarked the foundress marks, as social vertebrates do [53], [54]. Therefore, the colony odors of usurped nests became closer to the usurper odors than the matching control nests. When they emerged, *P. dominulus* workers from usurped nests learnt the usurper odor from their nests and tolerated their usurpers, whereas their sisters in control nests attacked usurpers, confirming that *P. nimphus* usurpers did not mimic, but overmarked host-nest odors [33].

There were two unexpected results when we analysed how *P. dominulus* workers from usurped nests responded: these workers accepted their mothers and erroneously accepted *P. nimphus* non-nestmates.

First, we can rule out the hypothesis that sensory modalities other than olfactory cues played a role in our experiments. Wasps use visual cues in nestmate recognition [11], [55], but workers in our experiment never met their foundresses or usurpers before recognition tests and had no clue other than scent marks to recognize them. It could be argued that *P. nimphus* wasps might bear visual (or olfactory) quality signals that inhibited attacks by *P. dominulus* workers. However, the workers in the usurped nests tolerated *P. nimphus* non-nestmates, but their sisters in the control nests attacked them, which suggested that quality signals did not affect workers' responses in our experiment. We can also rule out the hypothesis that *P. nimphus* usurpers added long-lasting appeasement substances to their half-nests, because *P. dominulus* workers were as aggressive towards conspecific non-nestmates as their sisters in control nests (whereas appeasement substances inhibit aggressive behaviors) [13], [56], [57]. Therefore, chemical recognition cues seem to mediate workers' responses in our experiment. Let's analyze recognition cues.

*P. dominulus* and *P. nimphus* had cuticular hydrocarbon profiles that differed only in their relative proportions of compounds, as they had matching chemical composition. Hence, when usurpers overmarked their half-nests, they only changed colony odors quantitatively. It may be argued that these quantitative changes were collectively small. However, they had large effects on worker nestmate recognition, suggesting that small quantitative changes played key roles in nestmate recognition.

The combined results of PCAs, DAs and behavioral tests suggested that colony odors variations in usurped nests consistently included increases in the discriminant peaks. We found that *P. dominulus* workers from usurped nests accepted their mothers, although our analyses showed that colony odor had changed in the usurped nests and workers learned usurper-biased colony odors. Workers from usurped nests might have recognized their genetic mothers if 1) workers learned their mothers' odors during pre-imaginal life, as they were fostered by their mothers during the larval stage [31], [58]; and/or 2) there were genetically encoded components in neural templates (as it occurs in fire ants and rodents) [3], [4], [8], [58], [59]. Future research will test these hypotheses. In contrast, we can rule out the possibility that workers learned their colony odors from themselves or from their nestmates [7], [31], since young social wasps have cuticular odors that differ from those of mature wasps [60].

*P. dominulus* workers from usurped nests erroneously accepted *P. nimphus* non-nestmates, but this was not due to the lack of individual odor variation between *P. nimphus* non-nestmates, so that these errors must be due to recognition errors. We noted that the discriminant peaks were significantly more concentrated in usurped nests than in control nests. This might have had consequences on the detection of odor differences. Indeed, the ability to perceive the difference between two stimuli depends on the magnitude of the stimuli themselves. Weber's Law of just noticeable difference (jnd) states that the jnd between two stimuli is proportional to the magnitude of the stimuli: the larger the magnitude of the stimuli, the larger the difference between the two stimuli, so that the difference is noticeable. This property is common to other sensory modalities, and may apply to olfactory stimuli as well, whenever, for example, doubling the concentration of the compared substance may lead to halving the discrimination power [61]. We can hypothesize that *P. dominulus* workers were unable to detect differences in hydrocarbon concentrations when the concentration in *P. nimphus* non-nestmates was beyond the range within which workers can easily discriminate differences. This suggests that *P. dominulus* workers recognized nestmates from non-nestmates on the basis of their relative proportions of hydrocarbons (as differences in relative proportions were the only differences between nestmate and non-nestmate odors), but only when the relative proportions varied within species-specific ranges. As a correlative support for this hypothesis, we found that worker discrimination abilities were the least in those colonies where the odor changes due to usurpation were the largest, suggesting that large increases in the concentrations of hydrocarbons triggered high tolerance towards any wasp of the other species, whereas less tolerance was measured in colonies where these increases were not as large.

From an evolutionary point of view, perceptual and learning abilities are expressed at the best within species-specific ranges, as they are shaped by the ecology of the species [62]. Anomalous stimuli are therefore less effective in shaping neural structures, which are pre-adapted to code for species-specific stimuli. Models on nestmate recognition mechanisms may need to incorporate the non-linear relationship between cue magnitude and response: for example, the acceptance threshold model [2], [63] assumes that social-insect guards evaluate the dissimilarity between the learned template and the recognition cues of unidentified individuals. Our results suggest that social insects might detect such dissimilarities only within species-specific ranges of hydrocarbon concentrations. Physiological tests are needed to test the hypothesis that hydrocarbon perception in social insects is non-linearly related to hydrocarbon concentrations, as suggested by our results.

Whatever the mechanism which might explain our results, we can infer some general speculation on the results of the behavioral tests.

One could argue that interspecific facultative parasitism could be much more common that actually is. Indeed, *Polistes* species are often sympatric and compete for nesting sites [64], [65]. Additionally, usurpers can easily mark usurped nests, change host-colony odor and trigger tolerance and cooperation by host workers. However, when interspecific usurpers increase the concentrations of some hydrocarbons, they may trigger tolerance not only towards themselves but also towards any wasp of their own species, according to our results. Eventually, these colonies are less defended against non-nestmates. This cost might act against the spread of interspecific facultative parasitism, especially in dense colony aggregations, where robbery among colonies may be common [66]. Our results also provide an interesting suggestion - a novel insight - on why specialized social parasites (i.e., obligate social parasites) might have been selected for mimicking, rather than marking, host colonies [13], [28], [67]. Host-nest overmarking may change social colonies into partially open societies, where some non-nestmates are erroneously accepted and resources easily stolen.
